# Multistate Outbreak of Human *Salmonella* Infections Linked to Live Poultry from a Mail-Order Hatchery in Ohio — March–September 2013

**Published:** 2014-03-14

**Authors:** Colin Basler, Tony M. Forshey, Kimberly Machesky, C. Matthew Erdman, Thomas M. Gomez, Thai-An Nguyen, Casey Barton Behravesh

**Affiliations:** 1 EIS Officer, CDC; 2 Ohio Department of Agriculture; 3 Ohio Department of Health; 4 US Department of Agriculture; 5 Division of Foodborne, Waterborne, and Environmental Diseases, National Center for Emerging and Zoonotic Infectious Diseases, CDC

In early 2013, four clusters of human *Salmonella* infections were identified through PulseNet, the national molecular subtyping network for foodborne bacteria. Many of the ill persons in these four clusters reported contact with live poultry, primarily chicks and ducklings, from a single mail-order hatchery; therefore, these investigations were merged. During March 4–October 9, 2013, a total of 158 persons infected with outbreak strains of *Salmonella* serotypes Infantis, Lille, Newport, and Mbandaka were reported from 30 states.

Forty-two percent (65 of 155) of ill persons were aged ≤10 years, and 28% (29 of 103) were hospitalized; no deaths were reported. Eighty-six percent (80 of 93) of ill persons who were interviewed reported live poultry contact in the week before illness onset. Sixty-nine percent (44 of 64) of ill persons who completed a supplemental live poultry questionnaire reported chick exposure, and 40% (26 of 64) reported duckling exposure. Seventy-five percent (33 of 44) of respondents reported live poultry exposure at their home; 59% (26 of 44) specifically reported keeping poultry inside their home.

Of the 40 ill persons who had recently purchased young poultry, the average time from purchase of poultry to illness onset was 21 days (range = 2–52 days); 48% (19 of 40) ill persons reported illness onset within 2 weeks of poultry purchase. Among persons with purchase information, 94% (62 of 66) reported buying young poultry sourced from a single mail-order hatchery in Ohio.

This outbreak investigation identified an Ohio hatchery as the likely source of the outbreak. This hatchery previously has been linked with multiple, large human *Salmonella* outbreaks (1,2). These recurring outbreaks highlight the need for comprehensive *Salmonella* prevention and control programs to be implemented and maintained at this mail-order hatchery and its associated breeder farms. Mail-order hatcheries and their source flocks should comply with management and sanitation practices outlined by the U.S. Department of Agriculture’s National Poultry Improvement Plan.* Additional owner education is necessary because healthy birds can still transmit *Salmonella* to humans. Educational material warning customers and advising them on how to reduce the risk for *Salmonella* infection from live poultry should be distributed by farm/feed stores and mail-order hatcheries with all live poultry purchases (3). Reducing the spread of *Salmonella* in mail-order hatcheries, in their source flocks, and in the feed store environment is critical to reduce the risk for human illness. This outbreak highlights the need for a comprehensive approach involving human and animal health officials and practitioners, industry, and backyard poultry flock owners.

## Footnotes

* Additional information available at http://www.aphis.usda.gov/publications/animal_health/content/printable_version/HelpingYouPoultryBreeder-PA1708-FinalJuly09.pdf.
